# Activity Level and Nature of Practice and Play in Children’s Football

**DOI:** 10.3390/ijerph19084598

**Published:** 2022-04-11

**Authors:** Tone Nybakken, Coral Falco

**Affiliations:** Department of Sport, Food and Natural Sciences, Western Norway University of Applied Sciences, 5020 Bergen, Norway; coral.falco@hvl.no

**Keywords:** deliberate play, deliberate practice, soccer, youth football, activity level

## Abstract

This study analyzes the activity level and nature of organized football training (deliberate practice, DPR), compared with when children play football on their own (deliberate play, DPL), in a sample of selected (YT) and non-selected (BT) talents. A total of 29 observations were analyzed over 2650 min, focusing on the kind of activity, variability, and intensity of the training. In DPL, there are more finishing on goal, involvement, and challenges in 1:1 situation, and more ball touches and ball transport in games, compared with DPR. Additionally, DPL has more activity time (68% vs. 56%) and fewer breaks overall (32% vs. 44%). In DPL, children spend more time playing against each other (92% vs. 36%), and most of the time there are games or finishing on goal. In DPR, children spend more time playing together with someone (2% vs. 44%) and in passing and receiving the ball. DPR training contains more standardized exercises and protected situations. DPR-YT training differs from DPR-BT training with less activity time, ball touches, attempts on goal, and 1:1 situations. In conclusion, the results support DPL providing more football-specific activity. More DPR training at the expense of DPL might reduce practice time for skill development.

## 1. Introduction

Children’s participation in organized soccer and the number of organized training sessions have increased in Norway in recent years [1]. The idea behind recruiting more children and increasing structured training environments in early ages is that the standard of soccer, in the long term, will improve. The Norwegian Federation of Sport (NIF) has provisions on child rights, highlighting that early specialization and early selection in sport should not occur [2]. Nevertheless, the field of practice is subject to influences that contravene the formal provisions and guidelines [3,4,5]. For example, the number of coaches for children and youth who are positive to early and specialized sports program has increased in Norway [4], as well as in other countries [6]. Players are selected for various teams and elite academies at an early age, this being considered important for the development of promising players [7,8,9]. Those sports programs appear to be characterized by early specialization and selection, elitism, and institutionalization, preventing children from participating in a diverse set of activities such as DPL, and requiring higher levels of investment from earlier ages [10,11].

It is unclear what models and developmental practice patterns best facilitate the development of elite performers. The theoretical framework of deliberate practice (DPR), the “early specialization way”, introduced by Ericsson, Krampe, and Tesch-Römer [12], is defined as highly structured activity that requires effort and systematic training with specialization and concentration in one sport. It contains clear and explicit rules, and the target focuses on the outcome rather than inherent enjoyment. Trainer-organized activity and influence are common. Ericsson et al. [12] argued that early specialization and 10,000 h of DPR was necessary for future success in becoming an expert, and that it would be next to impossible for a late starter to overcome the advantages of an early starter and the high amount of DPR training. Previous studies supporting the “early specialization way” have shown that experts were involved in higher numbers of sport-specific training (DPR) from an early age compared with non-experts [12,13,14,15,16,17,18,19,20]. However, these studies have been criticized for a monotonous relationship between the numbers of hours of DPR which individuals have performed [21], and/or for not exploring the connection with other factors (e.g., deliberate play, organized competitions and other sports) that are linked to sports participation in an individual developing perspective [22,23].

**Figure 1:**
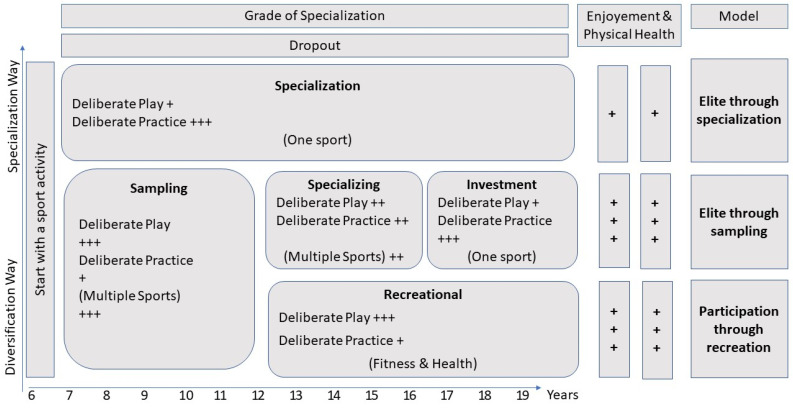
Developmental Model of Sport Participation Building on Côtè & Frasher-Thomas, 2007.

Contrary to DPR and the “early specialization way”, Côtè et al. [23,24,25] proposed deliberate play (DPL) and the “early diversification way” as an alternative in development to achieve the level of expertise. DPL activity is characterized as versatile and diversified sports practice and playful activity where interest is focused on the actual deployment. DPL includes classic self-organized neighborhood pickup games such as park football, which is usually played with small-sided teams and flexible peer-defined rules [24]. DPL activities involve an engagement of time in physical activities which is difficult to match in any kind of structured practice [26]. The DPL approach leads to positive motivation outcomes and increases a wide repertoire of transferable motor skills [6]. According to the Developmental Model of Sport Participation (DMSP) and the “early diversification way” (Figure 1), athletes should pass through three phases (sampling years, age 6–12; specializing years, age 13–15; investment years, age 16+) during their development in sport [25]. Those phases are continuous, which is applicable across high volumes of participation in DPL and involvement in various sports, with less structured and organized training to progressively decrease the participation in multiple sports and DPL, in favor of higher specialization, and DPR training [23,25,27].

Research evidence from team sports (e.g., hockey, field-hockey, basketball, and netball) suggests that a combination of DPR and DPL may significantly contribute to developing sport expertise [25,28,29]. Different studies have shown that athletes who have become elite in adulthood were involved in more playful, versatile activity training and several supplementary sports during childhood, compared with non-elite athletes. They also increase the number of trainer-oriented sports (DPR) later in their career [24,27,28,29,30]. Studies in football have shown that professional players were involved earlier and had accumulated more hours in both self-organized (DPL) and organized football training (DPR) than non-professionals [31,32,33]; moreover, specialization occurred later in their careers [33]. In addition, there is evidence that youth football players with professional contracts had spent more than double their time engaging in DPL activities in the sampling years compared with those without contracts [34,35]. Studies have also shown that only about 10% of early specialized football players progressed to professionals [32,36]. Mixed versions of these framework have also been promoted, such as the “early engagement pathway”, which is a less extreme version of specialization, and participation in DPL activities [32,37].

The developmental practice patterns for children in football are inconsistent [38], and the effects of DPR and DPL remain unclear [39]. Most of the studies in the field of DPL and DPR have been with elite athletes’ retrospective recall to compare successful performers [40]. The problem with retrospective studies is that they are difficult to remember, or the information is not accurate. It is not possible to make inferences, nor generalize on the part of the participants [37]. Moreover, studies that have analyzed DPL and DPR activities have focused on the number of hours involved, throughout adolescence and later in the career [6,7,12,13,14,15,17,18,19,23,25,27,28,30,31,33,34,37,41,42,43,44], rather than recording and analyzing well-defined activity types.

There is a lack of research regarding the content and possible differences in what players do in self-organized training compared with organized training. To the best of our knowledge, the DPL and the DPR content differences in the sampling years in football have not been extensively reported. It is important to understand the role of DPL and DPR in children’s football, especially because practice shows that this period coincides with an increase in DPR training, and the selection of players for academies and talent teams. Therefore, the purpose of this study was to investigate the activity level and nature in organized football training (DPR) compared with self-organized football activity (DPL) in a sample of young football players from a selected young talent team (YT) and non-selected talent from a base team (BT). We hypothesized that children playing football on their own (DPL) spend more time in game-like situations, are exposed to more ball touches, variability, and intensity compared with their DPR training. Additionally, the selected young players (YT) will show superior dominance in these variables compared with the non-selected players (BT). These analyses allow researchers to extract behavior patterns suitable for use by coaches to plan overall training during sampling years.

## 2. Materials and Methods

### 2.1. Participants and Procedures

A total of 29 observations of young athletes from two local football teams (at two different levels) were analyzed in both organized (deliberate practice, DPR, *n* = 16) and self-organized (deliberate play, DPL, *n* = 13) sessions. Boys aged 11 to 12 years voluntarily participated with informed consent from their parents. The study was approved by the ethical committee of the University and the Norwegian Center for research data (NSD). All participants had been playing football for six years with the same club and were divided into different talent levels (team for selected young talents, YT, and base team for non-selected talents, BT) by their sports club, when they turned 10 years old. This affected their time spent in organized and self-organized football activity, with less self-organized football for all, and more organized football for the best level players (YT). Ten observations of young athletes were analyzed in the team for non-selected talents (DPR-BT); six observations in the team were performed for selected talents (DPR-YT); and thirteen observations of young athletes were self-organized (DPL) in a small-sized pitch binge, where all players participated. The study observed the same young athletes in different arenas (DPR versus DPL—the same children). The study had three conditional levels: DPL, DPR-YT, and DPR-BT.

The training sessions observed were recorded on video, and randomly selected within a period of two months in the middle of the season. The DPL training sessions were observed the same week as the DPR training sessions to increase the probability that the motivations of the players and physical capacity were at the same level. Two video cameras were used for overall views and zooming situations, to cover all angles of the pitch. Test recording and analyses were conducted before the survey started. To analyze all the training, an ad hoc observational tool was created using a combination of the categorical system and format field. The definitions of the criteria, categories, and the variables, forming the observational tool (kind of activity, variability, and intensity) were developed considering the basic educational system of the football trainers as described by the Norwegian Football Federation (NFF) [1]. Following the recommendations made by Anguera et al. [45], passive observations, followed by several meetings, were held separately with two football experts to develop the categories: these were a national coach G 16–17 and chef of the national teams in Norway. Two experts in observational methodology were also consulted to assess the correct adjustment of the observational tools. Two observers were trained in both category recognition and codification, maintaining homogeneity of the inter- and intrasession and consistency of the observational periods [46]. The reliability of the registration codification derived by the observers was confirmed by: (a) a qualitative approach through consensual agreement [47]; and (b) calculating inter- and intra-observer agreement (intraclass coefficient correlations).

The video films were transferred and digitally compressed to Windows Media Player Audio (video file) and were analyzed on the PC with the Media Player Classic HC (It is a copyrighted, open source, and freely distributed software: https://github.com/mpc-hc/mpc-hc accessed on 27 November 2021) program. The data were encoded, analyzed, and quantified in an Excel file. Registration consisted of observing three main criteria: (1) the kind of activity; (2) variability; and (3) intensity, as well as the number of cases and time spent in different activities. These criteria and the descriptions of the associated 10 categories and 19 variables are presented in Table 1.

### 2.2. Analysis

Due to the different durations of the training sessions (80–140 min), time-average values were used to obtain comparable results. Registrations were divided into 5 min blocks, and blocks under 2.30 min were excluded from the analysis. Statistical analyses were carried out with SPSS version 26 (IBM Corp., Armonk, NY, USA). For all variables, the intra-class coefficient correlation was over 0.75 [48], showing a good reliability inter- and intra-observer. For categorical variables, contingency tables and Pearson’s standard chi-squared (χ^2^) was used to determine significant differences between conditions. For continuous variables, t-tests, and analysis of variance (ANOVA) was used to analyze differences between groups, mixing up independent group comparisons (DPR-BT and DPR-YT) and dependent samples (DPR versus DPL—the same children). Bonferroni adjustment for multiple comparisons was used to examine subsequently significant effects. When significant differences were obtained, partial eta squared (η^2^, for continuous variables), Cohen’s d scores (d) and Phi (φ) were quantified to analyze the effect size of the comparisons. η^2^ values below 0.01, 0.01–0.06, 0.06–0.14, and >0.14 were considered to have trivial, small, medium, and large effect sizes, respectively. A Cohen’s d value >0.8 indicated a large effect; 0.8–0.5, a moderate effect; 0.5–0.2, a small effect; and <0.2, a trivial effect. A Phi value >0.5 indicated a large effect; 0.49–0.30, a moderate effect; 0.29–0.10, a small affect; and <0.10 a trivial effect [49].

## 3. Results

The results are presented and analyzed between DPR and DPL, and on condition level (DPR-YT, DPR-BT and DPL), by (1) the kind of activity, (2) variability, and (3) intensity. The total observation time was 2650 min. Overall, 18,020 data values were collected, and 530 five-minute blocks were registered and analyzed on 29 observations of young athletes.

### 3.1. Kind of Activity 

Chi-squared analysis showed differences (χ^2^_(4)_ =178.8, *p* = 0.001, φ = 0.58) between DPL compared with DPR in the way football players used their total training time (see Table 2). 

Regarding the time spent training with a goal, there were significant differences (χ^2^_(1)_ =104.2, *p* = 0.001, φ = 0.44) between DPR and DPL, wherein DPR involved training with goals 51.9% of the time. In contrast, DPL involved playing with goals 93.9% of the time. 

Regarding playing in small and big groups (Category III), results showed significant differences (χ^2^_(2)_ = 254.5, *p* = 0.001, φ = 0.70) between DPR and DPL, i.e., DPR involved 21.4% and 14.5% in small and big groups, respectively. The other 64.2% of the time was devoted to other exercises. DPL incorporated 92.0% and 0.0% of the time in small and big groups, respectively. The other 8.0% was devoted to other exercises.

There were significant differences (χ^2^_(8)_ = 258.4, *p* = 0.001, φ = 0.70) between DPR-YT, DPR-BT, and DPL in the way they used their time during training. The results are presented in Table 3.

Regarding the use of goal during training, there were significant differences (χ^2^_(2)_ = 104.5, *p* = 0.001, φ = 0.44) between the three groups, i.e., DPR-YT involved 50.0% and 50.0% of the total training time playing with and without goals, whereas for DPR-BT, the proportion was 53.1% to 46.9%, respectively. In DPL, the percentages were 93.9% and 6.1%.

Regarding the time playing in small and big groups, there were significant differences (χ^2^_(2)_ = 386.3, *p* = 0.001, φ = 0.85) between the three groups, i.e., DPR-YT played in small groups 11.3% of the time and in big groups 37.1% of the time; the remaining 51.6% of the time was spent in other exercises. In DPR-BT, they played in small groups 27.8% of the time and 0.0% of the time in big groups; the remaining 72.2% was spent in other exercises. DPL spent 92.0% and 0.0% of the time in playing in small and big groups, respectively. The remaining 8.0% were used in other exercises.

### 3.2. Variability

#### 3.2.1. Ball Touches

Overall, 8878 ball touches were registered. No significant differences in ball touches (t = 0.34, *p* = 0.74) were found between DPR (M = 16.6) and DPL (M = 17.0) overall, on average, per every 5 min played. In game situations (category III), there were significant differences in ball touches (t = 3.55, *p* = 0.001, d = 0.8) between DPR (M = 12.6) and DPL (M = 19.3). When analyzing the three groups, the results showed significant differences between groups (F = 20.1, *p* = 0.001, η^2^ = 0.07). Subsequent comparisons showed that DPR-BT (M = 21.0) had more ball touches than DPL (M = 17.0) and DPR-YT (M = 9.7). In addition, DPL had more ball touches than DPR-YT. In game situations (category III), there were significant differences (F = 26.1, *p* = 0.001, η^2^ = 0.16) between groups, i.e., DPL (M = 19.3) and DPR-BT (M = 16.0) had more ball touches than DPR-YT (M = 9.6). No differences were found between DPR-BT and DPL (*p* > 0.05).

#### 3.2.2. Ball Variables

There were differences in passing (t = 6.0, *p* = 0.001, d = 0.6), receiving (t = 3.9, *p* = 0.001, d = 0.4), and transporting (t = 3.5, *p* = 0.001, d = 0.3) the ball between DPR and DPL. Specifically, DPR passed (M = 6.5), received (M = 5.2) and transported (M = 1.8) the ball more times than DPL (M = 2.5, M = 2.8, M = 0.6, respectively), on average, per 5 min played. In game situations (category III), the results showed significant differences between DPR and DPL in passing (t = 3.6, *p* = 0.001, d = 0.4) and transporting (t = −6.2, *p* = 0.001, d = 0.8), but not in receiving (t = 0.4, *p* = 0.68) the ball. Specifically, DPR passed (M = 4.0) the ball more times than DPL (M = 2.8), but transported the ball (M = 0.1) less than DPL (M = 0.7). No differences were found in receiving the ball between DPR and DPL (*p* > 0.05).

There were significant differences between DPR-YT, DPR-BT, and DPL, in passing (F = 39.1, *p* = 0.001, η^2^ = 0.13), receiving (F = 29.8, *p* = 0.01, η^2^ = 0.10), and transporting (F = 8.9, *p* = 0.001, η^2^ = 0.03) the ball. Subsequent Bonferroni multiple comparisons showed that DPR-BT (M = 8.6) passed the ball more times than DPR-YT (M = 3.3) and DPL (M = 2.5), but no differences were found between DPR-YT and DPL. Additionally, DPR-BT (M = 7.2) received the ball more times than DPR-YT (M = 2.1) and DPL (M = 2.8), but no differences were found between DPR-YT and DPL. Lastly, DPR-YT (M = 2.4) transported the ball more times than DPL (M = 0.6). No differences were found between DPR-BT (M = 1.4) and DPL, nor between DPR-YT and DPR-BT. In game situations (category III), the results showed significant differences in passing (F = 7.0, *p* = 0.001, η^2^ = 0.05), receiving (F = 4.5, *p* = 0.012, η^2^ = 0.03), and transporting (F = 19.5, *p* = 0.001, η^2^ = 0.13) the ball. Subsequent Bonferroni multiple comparisons showed that DPR-BT (M = 4.2) passed the ball more times than DPL (M = 2.8), but no differences were found between DPR-YT (M = 3.7) and DPL, nor between DPR-YT and DPR-BT. DPR-BT (M = 4.5) received the ball more times than DPR-YT (M = 2.8). No differences were found between DPR-BT compared with DPL (M = 3.4), nor between DPR-YT compared with DPL. Lastly, DPL (M = 0.7) transported the ball more times than DPR-YT (M = 0.1) and DPR-BT (M = 0.2). No differences were found between DPR-YT and DPR-BT (*p* > 0.05).

#### 3.2.3. Finishing on Goal

When it comes to finishing on goal, there were significant differences between DPR and DPL, (t = −6.2, *p* = 0.001, d = 0.6). Specifically, DPR (M = 1.0) attempted a shot at goal fewer times than DPL (M = 1.9), on average per 5 min played. In game situations (category III), the t-test (t = 6.7, *p* = 0.001, d = 0.9) showed that DPR (M = 0.8) attempted a shot at goal fewer times than the DPL (M = 2.1). Additionally, there were significant differences between the three groups (F = 38.5, *p* = 0.001, η^2^ = 0.13). Bonferroni multiple comparisons showed that DPL (M = 1.9) finished on goal more times than DPR-BT (M = 1.4) and DPR-YT (M = 0.3). DPR-BT also attempted a shot at goal more times than DPR-YT. In game situations (category III), the results showed significant differences between the three groups (F = 32.0, *p* = 0.001, η^2^ = 0.19). Concretely, Bonferroni multiple comparisons showed that DPL (M = 2.1) attempted a shot at goal more times than DPR-BT (M = 1.4) and DPR-YT (M = 0.3). DPR-BT also attempted finishing on goal more times than DPR-YT.

#### 3.2.4. 1:1 Situation

There were significant differences between DPR and DPL (t = −10.4, *p* = 0.001, d = 0.8) in 1:1 situations. Specifically, DPR (M = 2.1) were in 1:1 situation fewer times than DPL (M = 5.8), on average per 5 min played. In game situations (category III), there were also significant differences between DPR and DPL (t = −9.77, *p* = 0.001, d = 0.5). Specifically, DPR (M = 5.5) were in 1:1 situations fewer times than DPL (M = 7.4). In addition, there were significant differences between DPR and DPL (t = −8.9, *p* = 0.001, d = 0.7) in challenges with the ball in 1:1 situation. Specifically, DPR (M = 0.2) were involved in fewer challenges than DPL (M = 1.5). In game situations, there were also significant differences (t = −8.9, *p* = 0.001, d = 0.6) between DPR (M = 0.6) and DPL (M = 1.5) in challenging with the ball.

There were significant differences between DPR-YT, DPR-BT, and DPL in 1:1 situations (F = 54.4, *p* = 0.001, η^2^ = 0.17). Bonferroni multiple comparisons showed that DPL (M = 5.8) were more often involved in 1:1 situations than DPR-BT (M = 2.1) and DPR-YT (M = 2.1), but no differences were found between DPR-BT and DPR-YT. In game situations (category III), the results showed significant differences between groups (F = 16.1, *p* = 0.001, η^2^ = 0.11). Bonferroni multiple comparisons showed that DPL (M = 7.4) were more often involved in 1:1 situations than DPR-YT (M = 4.0), and DPR-BT (M = 7.1) were more often involved in 1:1 situations than DPR-YT. No differences were found between DPL and DPR-BT (*p* > 0.05). 

In addition, one-way ANOVA showed significant differences between DPR-YT, DPL-BT, and DPL (F = 39.2, *p* = 0.001, η^2^ = 0.13) in challenging with the ball in 1:1 situations. Bonferroni multiple comparisons showed that DPR-YT (M = 0.5) and DPR-BT (M = 0.8) were involved in fewer challenges than DPL (M = 1.5). There were no significant differences between DPR-YT and DPR-BT. In game situations, there were also significant differences between DPR-YT, DPR-BT, and DPL (F= 11.2, *p* = 0.001, η^2^ = 0.08) in challenging with the ball in 1:1 situation. Bonferroni multiple comparisons showed that DPR-YT (M = 0.3) and DPR-BT (M = 0.2) were involved in fewer challenges than DPL (M = 1.2). There were no significant differences between DPR-YT and DPR-BT (*p* > 0.05).

### 3.3. Intensity 

There were differences between DPR and DPL in activity time (t = −4.8, *p* = 0.001, d = 0.4), and in break time (t = 5.05, *p* = 0.001, d = 0.5). Specifically, DPR had less active time (M = 166.7) and more break time (M = 129.0) than DPL (M = 200.9 and M = 94.3, respectively), on average per 5 min played. In game situations, there were significant differences between DPR and DPL in activity time (t = −14.7, *p* = 0.001) and in time spent on breaks (t = 14.7; *p* = 0.001), i.e., DPR had less activity time (M = 134.4) and more break time (M = 159.4) than DPL (M = 222.7 and M = 74.0, respectively).

One-way ANOVA showed significant differences between groups in activity time (F = 15.5, *p* = 0.001, η^2^ = 0.06) and break time (F = 15.2, *p* = 0.001, η^2^ = 0.06). Subsequent Bonferroni multiple comparisons showed that DPR-YT (M = 150.9) had less activity time than DPR-BT (M = 176.4) and DPL (M = 200.9), as well as DPR-BT compared with DPL. Additionally, DPR-YT (M = 140.9) and DPR-BT (M = 121.5) demonstrated more break time than DPL (M = 94.3). No differences were found between DPR-YT and DPR-BT (*p* > 0.05).

In game situations, one-way ANOVA also showed significant differences between groups in activity time (F = 6.5, *p* = 0.002, η^2^ = 0.05) and in break time (F = 6.1, *p* = 0.003, η^2^ = 0.04). Subsequent Bonferroni multiple comparisons showed that DPR-YT (M = 200.9) had less activity time than DPR-BT (M = 242.8) and DPL (M = 224.2). There were no differences between DPR-BT and DPL. Lastly, DPR-YT had more break time (M = 94.5) than DPR-BT (M = 55.2). No significant differences were found between DPR-YT and DPL (M = 72.7), nor between DPR-BT and DPL (*p* > 0.05).

## 4. Discussion

This study investigated activity the level and nature of organized football training (deliberate practice, DPR), compared with when the same children played football on their own (deliberate play, DPL). It also included a comparison between selected young talents (YT) and non-selected talents (BT), and the kind of activity, variability, and intensity in DPR (DPR-YT, DPR-BT) and DPL training. Most of the results showed a moderate to large effect size. To the best of our knowledge, this is the first study which has analyzed and compared the content in activities developed during DPR and DPL training, as well as the intensity and duration of the sessions, and by observing the same children in different arenas. 

The main results of this study showed that players in DPL spent more time playing against each other (Category III, games), attacking the goal, playing in small groups, and had more activity time and fewer breaks, compared with DPR. Furthermore, DPL had significantly more finishing on goal, involvement, and challenges in 1:1 situations, as well as ball touches and ball transport in games, compared with DPR. In contrast, DPR spent most of the time playing together with someone (Category II), and in set situations for receiving and passing the ball. Selected young talent training sessions (DPR-YT) involved the most breaks, fewer ball touches, fewer finishing on goal, as well as fewer 1:1 situations and less activity time in games. Finally, DPR-BT training stood out with most time spent in playing together with someone (Category II), and the most ball touches, passes, and receiving, related to standardized exercises that usually are carried out during training sessions. 

### 4.1. Kind of Activities

Regarding the kind of activity, the players in DPL were exposed to more complex and varying training situational contexts, spending more than double the time (92% vs. 36%) in games (Category III), and nearly double the time playing with goals, as compared with DPR (also DPR-YT). The DPR training in the present study is not in line with previous studies that have highlighted the importance of replicating the game conditions during training [41,50]. Interestingly, DPR-YT spent more of their time in big groups, whereas for DPR-BT, this was the opposite. Unlike DPL, DPR can play in larger groups (full match team), because they normally play games with more players on the field. On the other hand, it is a deliberate strategy of the NFF to play matches with fewer players for children; such as 5-a-side and 7-a-side football, because it increases the possibility of more ball touches and involvement for each player [51]. Our results in DPR are in line with other research in the field, which found that youth players in DPR training spent more time in enhancing less soccer-match-relevant performance (e.g., technique of passing and skills practices, physical training), than activities more relevance, such as small-sided/conditioned games and phase-of-play activities [52]. Versatile playing forms of activity are assumed to provide more “effective development of the perceptual, cognitive and technical skills underpinning superior performance” [41]. Training with small-sided games and match play are practices that can be linked to random, variable practice conditions and frequent situational repetitions [50]. The interactions between perceptual, cognitive, and motor skills required for a match are difficult to replicate in drill-type exercises [52]. A large predominance of training in Category II indicates that the DPR training was a more controlled, standardized, drill-like exercise, with the blocked, constant practice of a single skill. DPR training may indicate training with the use of more constraints and in more protected situations from external stress from the environment or the task [53,54], such as opposing players and complex player situations. This may also be necessary in some phases of development, but the lack of major challenges may be negative for development in the long term. Training practice in the different categories mentioned is associated with different benefits. This is in line with Williams and Hodges [50], who stated that random practice is more effective for skill learning, whereas specific and blocked practice is better for improving performance. Interestingly, Ericsson et al. [12] argued that a large amount of DPR training will make athletes better able to cope with stress. Our results do not indicate this, because the players were more in complex game situations in DPL. To be able to mimic the complex environment and the variation experienced during a match in training, this will probably mean that coaches are able to incorporate more variable practice conditions, as opposed to more traditional coaching [50].

### 4.2. Variability

When it comes to variability, the results showed that training in DPL compared with DPR involves more practice with ball variables and in match-like and close-up situations than is found in natural football matches (e.g., more attacking, finishing on goal, ball touches, 1:1 situations, challenges, and ball transport in games). In line with Sagar and Lavelle [55], who found that player mistakes in adult-led practice can be appraised by young players to be a threat and feared, players may not dare to be as creative and try out new skills in DPR as in DPL. Overall, DPL passed, received, and transported the ball fewer times than DPR. Moreover, in game situations, there were more ball transport and ball touches in DPL, double that in DPR. Regarding conditional levels, DPR-BT showed the highest proportion of ball touches, which was due to their longer time spending in warm-up exercises with ball, and in set situations for passing and receiving the ball. DPR-BT also showed more finishing on goal, 1:1 situation, in games, compared with DPR-YT. When the selected talents (YT) played football in DPL, they had twice as many ball touches, six times more finishing on goal, and three times more involvement and challenges in 1:1 situations, as in organized football training (DPR-YT). This indicates that the players have less variability in their DPR training (YT in particular), compared with DPL. It seems that in DPR training, players do not explore their physical capacities in various contexts, or experienced enough variability in practice or discovery learning, which is important from a motor skill acquisition perspective [53,56]. In line with the developmental model DMSP and the “early diversification way”, our study highlights the importance of DPL training to include the variability needed early to develop emotional, cognitive, and motor skills which will provide benefits in their later sports career [6,25]. This could be explained from the view that play, unlike exercise, involves more variable interactions and several similar exercises but in different action situations, which contributes to the development of adaptable transferable skills and extensive implicit skill learning [28,33,50]. One can speculate that players do not choose those exercises they find boring in DPL, which overall can be useful to provide even better ball control and variety. Games were preferred when players could decide for themselves, which may indicate that they found the game situations as the most motivating in football. This is also supported by other researchers such as Côtè et al. [6], who claimed that DPL leads to positive motivation outcomes. Therefore, playful activities are of importance to stimulate players’ intelligence and contribute to perception and decision making in football games [57], and contributes to the development of creativity [58]. DPL facilitates opportunities for individual and experimental varying technical and tactical solutions, and provides improved behavior and nerve connections, developing patterns when children’s development occurs, thus helping to maintain technique in stressful situations [54].

### 4.3. Intensity

An important finding in the present study was that in DPL training, the activity time was higher and contained fewer breaks (both in exercises and between exercises), compared with DPR. This applied to the entire training and in games, but the differences increased further in games even though the effect size was small. Selected young talent training (DPR-YT) had less activity time than DPL and DPR-BT, as well as more breaks than DPL overall and DPR-BT in games. There were no significant differences in overall breaks between DPR YT and DPR-BT. Our results are in line with previous research in the field [59,60,61], wherein athletes’ time spent on tasks (activity time) in structured practice (DPR) varied between 25% and 54% of the total practice time [26]. In our study, we found that in DPR, 56% of the total time was spent being active, and in DPL, 68% of the total time was spent being active. Previous research (e.g., in basketball), also found fewer periods of waiting or off-tasks in DPL basketball compared with structured basketball training [26]. Based on the higher percentage of DPL active time compared with DPR, it might be possible that the athletes wish to be more active in DPR training than they are permitted. The difference might also be natural, because trainers use breaks to give instructions, feedback, and explanations during DPR training. The question is, however, if those pauses are too long because the results showed longer pauses both in and between exercises. It might be expected that the YT training had more active time.

A surprising result was that the main differences (number of different ball touches, variability, and intensity) were between DPR-YT and DPL, and not between DPR-BT and DPL, as might be assumed. Even if DPR-YT transported the ball more, the number dropped considerably in game situations, which may indicate that the players dared and challenged less in complex situations in DPR-YT, compared with DPL. Notably, the selected talents seemed to be offered the narrowest training environment (such as fewer ball touches, fewer finishing on goal, fewer 1:1 situations, and fewer match-like and complex situations). In the long term, this could be negative for the development of motor skills and talent realization. On the other hand, they might be able to play with better players in selected teams, which may increase the quality of training. The selection itself can also give the chosen players a boost [62]. Hence, our findings indicate advantages in soccer to include DPL in training as well as DPR; activity in DPL does not necessarily increase the ability to fit into interactions with complete player pattern in a team. DPR, unlike DPL, has advantages in that coaches are able to provide feedback and instructions [50]. It is also possible that DPL provides a greater advantage for the best players, to exert control and emphasize themselves more, at the expense of inferior players. However, and in line with Cotê et al. [6], it is unclear whether the benefits in DPR are superior to the benefits in DPL, or the opposite.

Considerable research has shown that players who have reached elite level in football had substantial DPL involvement during childhood and youth, in football-specific play activities [37,44,63], together with DPR in varying amounts [31,33,34,57]. Although it has been argued that DPR training contains elements of play activity, Ford et al. [52] showed that only a small part of the training was emphasized for playing time form in DPR (about 30% of the training among 9-year-olds, and about 40% among 13-year-olds). Our results add to the growing body of evidence supporting the need for DPL as a contribution in children’s training (during sampling years). It seems that the children had more skill practice in DPL, whereas in DPR, there was more performance training. Based on the findings in the present study, one can hardly see that DPR includes a greater variability of practice, as compared with DPL. Rather, it appears that children have a greater variability and allow for more discovery learning in self-organized activities. A higher number of organized training sessions at the expense of self-organized activity might be negative for the quality of talent development, unless sufficient DPL variability is adapted into DPR.

The present study has five limitations. The small sample size, and the fact that this study only included two teams, means that the results are not representative of all countries; therefore, the study cannot be generalized to the Norwegian population. Second, the study contained only one season and could not predict the success of the athletes. Third, we cannot ignore the fact that training included sprint, jogging, and walking time. In the present study, we have not analyzed those variables separately, and they may show differences regarding the intensity of the training. Additionally, the effect of early diversification (multiple sports) was not studied. Lastly, the study does not consider the quality various exercises and the leadership level of the coaches. Therefore, future research should include these variables. However, to be able to understand the practice and play activities performed by team sport players, more research examining in microstructure is needed.

### 4.4. Practical Implications

Acquiring more knowledge about DPR and DPL is of interest, because it has implications for training offers, talent identification, and the management of sports clubs. A sports club strategy with an increased number of training hours in DPR and talent schools, at the expense of DPL training, can lead to less mature and trained players (late bloomers) being kept out of football. How managers and coaches understand the concept of talent, as something that is static (some have talent and others do not) or dynamic (development and changes over time), may affect how they facilitate the overall training environment. To date, there has been no test battery developed which identifies a good performer in the long term [64,65]. The general assumptions and increasing trends and strategies in Norway have been to involve more children in DPR practice early on. Based on the current practice, it may appear that athletes or children who develop their skills alone or with others in DPL activity, without coaches present, are not sufficiently valued in football. It may be helpful for the development of skills to encourage children to be more involved in the DPL learning context in early years. However, it is not clear whether all forms of DPL are of equal value. In the future, researchers should focus more on the content and quality of the activity than on the number of hours spent in DPL and DPR.

## 5. Conclusions

Four main conclusions can be drawn from the present study. First, self-organized (DPL) and organized soccer (DPR) activities are very different in many respects. The activity level, the intensity, and variability in the activities are higher in DPL as compared with DPR situations.

Regarding the kind of activity, in DPL, players spend more time playing against each other (games), having more finishing on goal and more playing in small groups, as compared with DPR. In contrast, DPR, and especially DPR-BT, involves more time playing together with someone (exercises without an opposing team). Second, when it comes to variability in the training, DPL training is characterized by having more ball touches and ball transport in games; overall, there are also more 1:1 situations, challenges, and attempts on goals, in an exploratory environment. In contrast, in DPR training, most of the time spent in set situations is for receiving and passing the ball, and in standardized exercises with more constraints and exercises of a single skill. Third, in contrast to DPR-YT, DPR-BT had a higher number of ball touches, passes, and received the ball more, attempted more shots and headings on goal, and were more often involved in 1:1 situations in games. Selected young talents (YT) had twice as many ball touches and more variability in DPL as in their DPR-YT training. Lastly, DPL had more effective activity time and considerably fewer breaks than DPR. DPR-BT also had more activity time than DPR-YT.

## Figures and Tables

**Table 1:** An overview of registration on criteria, categories, and variables.

Criteria	Categories	Variables	Description
(1) Kind of activity	I: You and the ball	(1) Training alone with the ball	All kinds of activity when you are alone with the ball, e.g., technique, tricking the ball, etc.
	II: Playing together with someone	(2) Playing with goals	All kind of playing with someone with goals involved in the exercise. No opposing players or team.
		(3) Playing without goals	All kind of playing with someone without goals, e.g., passes and receiving exercises. No opposing players/team.
	III: Play against each other	(4) Small group game with goals	Play against an opposing team ≤ 5 to 5, with goals.
		(5) Small group game without goals	Playing against an opposing team ≤ 5 to 5, without goals.
		(6) Big group game with goals	Play against an opposing team ≥ 6 against 5, with goals.
		(7) Big group game without goals	Play against an opposing team ≥ 6 against 5, without goals.
	IV: Other activity	(8) All activities without ball	Strength training, running exercise, stretching, playing, relay activity, etc.
	V: Breaks	(9) Breaks	No movement (in seconds) between exercises (e.g., water break, waiting for trainers to explain or start a new exercise, etc.).
(2) Variability	VI: Ball touches	(10) Ball touches	Total number of ball touches.
	VII: Ball variables	(11) Ball transport	Number of continuous ball touches ≥ 4 touches in a row.
		(12) Ball receiving	Number of 1–3 ball touches in receiving sequence.
		(13) Ball passes	Number of 1–3 ball touches in passes sequence.
		(14) Finishing on goal	Number of attacks on goal (shot and heading).
		(15) Other ball touches	Number of other types of ball touches, such as clearances, throw-in, ball restarts, etc.
	VIII: 1:1 situations	(16) 1:1 situations	Number of all 1:1 situations in attack and defense.
		(17) Challenge in 1:1 situations	Number of all the times they choose to challenge and try to pass a player in 1:1 situations.
(3) Intensity	IX: Activity time	(18) Activity time	All kinds of movement in training at maximum running speed (sprint), jogging and walking.
	X: Break time	(19) Total break time	Players stand still without moving. Total breaks during the exercise and between exercises (e.g., waiting to be involved/or self-involved in the exercise, water break, waiting for trainers to explain next drill, provides feedback, start a new exercise, etc.)

**Table 2:** Percentages of the time spent in different kinds of activities between deliberate practice and deliberate play, registered on the average rates per every 5 min played.

	I	II	III	IV	V	Total
	You and the ball	Play together with someone	Play against each other	Other activity	Breaks	100%
Deliberate Practice (DPR)	0.6%	44.0%	35.8%	6.9%	12.6%	100%
Deliberate Play (DPL)	2.4%	1.9%	92.0%	1.9%	1.9%	100%

**Table 3:** Percentages of the time spent in different kinds of activities, divided between DPR-YT, DPR-BT, and DPL, registered on the average rates per every 5 min played.

	I	II	III	IV	V	Total
	You and the ball	Play together with someone	Play against each other	Other activity	Breaks	
Deliberate Practice (DPR-YT)	1.6%	19.4%	48.4%	14.5%	16.1%	100%
Deliberate Practice (DPR-BT)	0.0%	59.8%	27.8%	2.1%	10.3%	100%
Deliberate Play (DPL)	2.4%	1.9%	92.0%	1.9%	1.9%	100%

## References

[1] Association N.F. Statistics and History, Number of Teams Overtime 2007 and 2017. https://www.fotball.no/tema/om-nff/statistikk-og-historikk/antall-lag-over-tid/.

[2] Federation N.S. Children’s Right in Sport. The Provisions on Children’s Sports. https://www.idrettsforbundet.no/contentassets/482e66e842fa4979902ecc77f0c05263/36_17_barneidrettsbestemmelsene_eng.pdf.

[3] Augestad P., Bergsgaard N.A., Houlihan B., Green M. (2008). Comparative elite sport development: Systems, structures and public policy—Norway. Comparative Elite Sport Development: Systems, Structures and Public Policy.

[4] Loland S. Evaluation of Ethics and Value Work in the Norwegian Sports Confederation. https://www.idrettsforbundet.no/.

[5] Ommundsen Y., Johansen B.T., Høigaard R., Fjeld J.B. (2009). Who are the talents, do we have to specialize early, and what is a good coach. Newer Perspectives in Sports and Sports Pedagogy.

[6] Côté J., Lidor R., Hackfort D. (2009). ISSP position stand: To sample or to specialize? Seven postulates about youth sport activities that lead to continued participation and elite performance. Int. J. Sport Exerc. Psychol..

[7] Ford P.R., Le Gall F., Carling C., Williams A.M. (2009). A cross-cultural comparison of the participation histories of English and French elite youth soccer players. Science and Football VI.

[8] Larkin P., O’Connor D. (2017). Talent identification and recruitment in youth soccer: Recruiter’s perceptions of the key attributes for player recruitment. PLoS ONE.

[9] Meylan C., Cronin J., Oliver J., Hughes M. (2010). Talent identification in soccer: The role of maturity status on physical, physiological and technical characteristics. Int. J. Sports Sci. Coach..

[10] Gould D., Carson S. (2004). Fun & games? Myths surrounding the role of youth sports in developing Olympic champions. Youth Stud. Aust..

[11] Hecimovich M. (2004). Sport specialization in youth: A literature review. J. Am. Chiropr. Assoc..

[12] Ericsson K.A., Krampe R.T., Tesch-Römer C. (1993). The role of deliberate practice in the acquisition of expert performance. Psychol. Rev..

[13] Ahmad M.F., Low J.F.L., Nadzalan A.M., Aziz A.A. (2020). Developmental Practice Activities of Youth Soccer Players. Eur. J. Mol. Clin. Med..

[14] Baker J., Cote J., Abernethy B. (2003). Learning from the experts: Practice activities of expert decision makers in sport. Res. Q. Exerc. Sport.

[15] Deakin J.M., Cobley S., Starkes J.L., Ericsson K.A. (2003). A search for deliberate practice: An examination of the practice environments in figure skating and volleyball. Expert Performance in Sports: Advances in Research on Sport Expertise.

[16] Helsen W.F., Hodges N.J., Van Winckel J., Starkes J.L. (2000). The roles of talent, physical precocity and practice in the development of soccer expertise. J. Sports Sci..

[17] Helsen W.F., Starkes J.L., Hodges N.J. (1998). Team sports and the theory of deliberate practice. J. Sport Exerc. Psychol..

[18] Hodges N.J., Starkes J.L. (1996). Wrestling with the nature expertise: A sport specific test of Ericsson, Krampe and Tesch-Römer’s (1993) theory of “deliberate practice”. Int. J. Sport Psychol..

[19] Law M.P., Côté J., Ericsson K.A. (2007). Characteristics of expert development in rhythmic gymnastics: A retrospective study. Int. J. Sport Exerc. Psychol..

[20] Ward P., Hodges N.J., Starkes J.L., Williams M.A. (2007). The road to excellence: Deliberate practice and the development of expertise. High Abil. Stud..

[21] Tucker R., Collins M. (2012). What makes champions? A review of the relative contribution of genes and training to sporting success. Br. J. Sports Med..

[22] Baker J., Young B.W., Tedesqui R.A., McCardle L., Tenenbaum G., Eklund R.C. (2020). New perspectives on deliberate practice and the development of sport expertise. Handbook of Sport Psychology.

[23] Côté J., Baker J., Abernethy B., Starkes J., Ericsson K.A. (2003). From play to practice: A developmental framework for the acquisition of expertise in team sports. Recent Advances in Research on Sport Expertise.

[24] Côtè J. (1999). The influence of the family in the development of talent in sports. Sport Psychol..

[25] Côté J., Baker J., Abernethy B., Tenenbaum G., Eklund R.C. (2007). Practice and play in the development of sport expertise. Handbook of Sport Psychology.

[26] Baker J., Young B. (2014). 20 years later: Deliberate practice and the development of expertise in sport. Int. Rev. Sport Exerc. Psychol..

[27] Côté J., Fraser-Thomas J., Crocker P.R.E. (2007). Youth involvement in sport. Sport Psychology: A Canadian Perspective.

[28] Baker J., Cote J., Abernethy B. (2003). Sport-specific practice and the development of expert decision-making in team ball sports. J. Appl. Sport Psychol..

[29] Soberlak P., Côté J. (2003). The Developmental Activities of Elite Ice Hockey Players. J. Appl. Sport Psychol..

[30] Gilberg R., Breivik G. (1999). Why did the Best Become the Best? Childhood, Adolescence and Athletic Development Among 18 of the Most Successful Norwegian Athletes.

[31] Haugaasen M., Toering T., Jordet G. (2014). From childhood to senior professional football: A multi-level approach to elite youth football players’ engagement in football-specific activities. Psychol. Sport Exerc..

[32] Hendry D.T., Hodges N.J. (2018). Early majority engagement pathway best defines transitions from youth to adult elite men’s soccer in the UK: A three time-point retrospective and prospective study. Psychol. Sport Exerc..

[33] Hornig M., Aust F., Güllich A. (2016). Practice and play in the development of German top-level professional football players. Eur. J. Sport Sci..

[34] Ford P.R., Ward P., Hodges N., Williams A.M. (2006). Antecedents of selection into professional soccer: The roles of play and practice in progression and regression. J. Sport Exerc. Psychol..

[35] Williams A.M., Ford P.R. (2008). Expertise and expert performance in sport. Int. Rev. Sport Exserc. Psychol..

[36] McLeish S., Collins D. (2001). Talent Identification and Development in Scottish Soccer: Where did we go Wrong?. Ph.D. Thesis.

[37] Ford P.R., Ward P., Hodges N.J., Williams A.M. (2009). The role of deliberate practice and play in career progression in sport: The early engagement hypothesis. High Abil. Stud..

[38] Güllich A., Emrich E. (2014). Considering long-term sustainability in the development of world class success. Eur. J. Sport Sci..

[39] Güllich A., Cronauer R., Diehl J., Gard L., Miller C. (2020). Coach-assessed skill learning progress of youth soccer players correlates with earlier childhood practice in other sports. Int. J. Sports Sci. Coach..

[40] Coutinho P., Mesquita I., Fonseca A.M. (2016). Talent development in sport: A critical review of pathways to expert performance. Int. J. Sports Sci. Coach..

[41] Cushion C., Ford P.R., Williams A.M. (2012). Coach behaviors and practice structures in youth soccer: Implications for talent development. J. Sports Sci..

[42] Fraser-Thomas J., Coté J., Deakin J.M. (2008). Examining adolescent sport dropout and prolonged engagement from a developmental perspective. J. Appl. Sport Psychol..

[43] Güllich A. (2017). International medallists’ and non-medallists’ developmental sport activities–A matched-pairs analysis. J. Sport Sci..

[44] Ford P.R., Williams A.M. (2012). The developmental activities engaged in by elite youth soccer players who progressed to professional status compared to those who did not. Psychol. Sport Exerc..

[45] Anguera M.T., Magnussson M.S., Jonsson G.K. (2007). Instrumentos no estándar: Planteamiento, desarrollo y posibilidades. Av. En Med..

[46] Anguera M.T., Blanco A., Hernández-Mendo A., Losada J.L. (2011). Diseños Observacionales: Ajuste y aplicación en psicología del deporte. Cuad. Psicol. Deporte.

[47] Usabiaga O., Castellano J., Blanco-Villaseñor A., Casamichana D. (2013). La Teoría de la Generalizabilidad en las primeras fases del método observacional aplicado en el ámbito de la iniciación deportiva: Calidad del dato y estimación de la muestra. Rev. Psicol. Deporte.

[48] Koo T.K., Li M.Y. (2016). A Guideline of Selecting and Reporting Intraclass Correlation Coefficients for Reliability Research. J. Chiropr. Med..

[49] Cohen J. (1988). Statistical Power Analysis for the Behavioural Sciences.

[50] Williams A.M., Hodges N.J. (2005). Practice, instruction and skill acquisition in soccer: Challenging tradition. J. Sports Sci..

[51] Association N.F. Guidelines for Children’s and Youth Football. https://www.fotball.no/barn-og-ungdom/retningslinjer-for-barne--og-ungdomsfotball.

[52] Ford P.R., Yates I., Williams A.M. (2010). An analysis of practice activities and instructional behaviours used by youth soccer coaches during practice: Exploring the link between science and application. J. Sports Sci..

[53] Vereijken B., Hopkins B., Barr R.G., Michel G.F., Rochat P. (2005). Motor development. The Cambridge Encyclopedia of Child Development.

[54] Thelen E., Smith L.B. (1994). A Dynamic System Approach to the Development of Cognition and Action.

[55] Sagar S.S., Lavallee D. (2010). The developmental origins of fear of failure in adolescent athletes: Examining parental practices. Psychol. Sport Exerc..

[56] Schmidt R.A. (1991). Motor Learning & Performance. From Principles to Practice.

[57] Berry J., Abernethy B., Cote J. (2008). The contribution of structured activity and deliberate play to the development of expert perceptual and decision-making skill. J. Sport Exerc. Psychol..

[58] Memmert D., Baker J., Bertsch C. (2010). Play and practice in the development of sport-specific creativity in team ball sports. High Abil. Stud..

[59] Boudreau P., Tousignant M. (1991). L’efficacite de l’intervention d’entraineurs benevoles en formation. Can. J. Sport Sci..

[60] Brunelle J., Spallanzani C., Tousignant M., Martel D., Gagnon J. (1989). Effets d’une stratégie d’auto-supervision sur les composantes du temps d’apprentissage dans l’enseignement de deux sports. Can. J. Educ..

[61] Wuest D., Mancini V., Mars H., Terrillion K. (1986). The Academic Learning Time-Physical Education of High- Average- and Low-Skilled Female Intercollegiate Volleyball Players. In Proceedings of the Sport Pedagogy: The Olympic Scientific Congress. https://www.researchgate.net/publication/273451870_17_-_The_Academic_Learning_Time-Physical_Education_of_High-_Average-_and_LowSkilled_Female_Intercollegiate_Volleyball_Players.

[62] Thompson A.H., Barnsley R.H., Battle J. (2004). The relative age effect and the development of self-esteem. Educ. Res..

[63] Roca A., Williams A.M., Ford P.R. (2012). Developmental activities and the acquisition of superior anticipation and decision making in soccer players. J. Sports Sci..

[64] Abbot A., Collins D. (2004). Eliminating the dichotomy between theory and practice in talent identification and development: Considering the role of psychology. J. Sports Sci..

[65] Bloom B.S. (1985). Developing Talent in Young People.

